# Deciphering the Enigma of Calcific Aortic Valve Disease: The Pivotal Role of Animal Models in Unraveling Pathogenesis and Advancing Therapeutic Strategies

**DOI:** 10.3390/biomedicines13102369

**Published:** 2025-09-27

**Authors:** Pengning Fan, Yuqi Liu, Xingyu Qian, Fuqiang Tong, Yidan Zheng, Zhengfeng Fan, Ming Chen, Zhe Chen, Haoyang Zhai, Teng Zeng, Nianguo Dong, Fei Li, Xucong Shi, Li Xu

**Affiliations:** 1 Department of Cardiovascular Surgery, Union Hospital, Tongji Medical College, Huazhong University of Science and Technology, Wuhan 430022, China; pengning_fan@163.com (P.F.); 2023xh5092@hust.edu.cn (Y.L.); deus23@foxmail.com (X.Q.); u201710306@hust.edu.cn (F.T.); u201910272@hust.edu.cn (Y.Z.); fanzhf16doct@163.com (Z.F.); jeremychen_med@163.com (M.C.); cz09150806@163.com (Z.C.); zhaihaoyang2@gmail.com (H.Z.); m202476307@hust.edu.cn (T.Z.); 1986xh0694@hust.edu.cn (N.D.); lifei_union@hust.edu.cn (F.L.); 2 Key Laboratory of Molecular Biological Targeted Therapies of the Ministry of Education, Huazhong University of Science and Technology, Wuhan 430022, China; 3 Department of Structure Heart Center, FuWai Yunnan Cardiovascular Hospital, Kunming 650021, China; 4 Department of Cardiac Surgery, Children’s Hospital, Zhejiang University School of Medicine, National Clinical Research Center for Child Health, Hangzhou 310058, China

**Keywords:** calcific aortic valve disease, animal model, in vivo, aortic valve calcification assessment

## Abstract

Calcific aortic valve disease (CAVD) is a prevalent cardiovascular condition and is the most common heart valve disease globally. Hyperlipidemia and aging are key risk factors; consequently, with the aging global population, CAVD incidence continues to rise. Despite extensive research, the pathogenesis of CAVD remains unclear, leading to a lack of effective pharmacological therapies. Consequently, valve replacement surgery persists as the primary treatment option. Establishing suitable animal models is crucial for investigating the complex pathophysiological mechanisms of CAVD in vivo, although an optimal model has yet to be identified. This review provides a concise overview of CAVD pathogenesis and summarizes the application of common animal models—including mice, rats, rabbits, and pigs—in studying valve calcification. We specifically detail the construction of various models and their associated calcific aortic valve phenotypes. Furthermore, we outline common detection methods for assessing aortic valve calcification in these models and suggest future directions for developing improved animal models relevant to CAVD research.

## 1. Introduction

Calcific aortic valve disease (CAVD) is a prevalent cardiovascular condition and globally the most common heart valve disease [1]. Recent epidemiological studies estimate that CAVD affects approximately 12.6 million individuals worldwide, resulting in 121,700 annual deaths [2]. CAVD development is associated with multiple risk factors, including age, hypertension, diabetes mellitus, obesity, elevated plasma lipoprotein levels, and bicuspid aortic valve [1]. Age is the primary risk factor, with prevalence doubling every decade [3]. Consequently, rising life expectancy is projected to increase the incidence and prevalence of CAVD.

The pathologic changes of CAVD are a continuous process. Initially, CAVD advances to aortic stenosis (AS), characterized histopathologically by increased fibrous matrix deposition and calcified nodule formation [4]. These alterations disrupt the normal leaflet architecture, compromise the aortic valve’s biomechanical properties, and ultimately lead to AS. Severe AS is frequently associated with life-threatening symptoms such as dyspnea, angina pectoris, and syncope [5]. Currently, prosthetic aortic valve replacement is the primary effective treatment for AS [6]. However, surgical risks, alongside structural deterioration or thrombosis of valve prostheses, limit its therapeutic applicability [7]. Consequently, the development of effective pharmaceutical interventions for CAVD has become a major research focus.

Elucidating the pathogenesis of CAVD is crucial for identifying novel therapeutic targets and exploring promising research avenues. CAVD arises from diverse stimuli, including lipid deposition, inflammation, oxidative stress, and abnormal hemodynamic forces, involving valve endothelial cells (VECs), valve interstitial cells (VICs), macrophages, and other immune cells [8,9,10]. The differentiation of VICs into osteoblast-like cells is now recognized as a pivotal event in CAVD progression [11]. CAVD research typically employs both in vitro and in vivo approaches. In vitro VIC culture serves as a widely used model for investigating calcification mechanisms at cellular and molecular levels, whereas in vivo animal models are essential for exploring pathogenesis within the complex pathophysiological environment [12,13]. Although no single animal model fully recapitulates human CAVD, ongoing research focuses on enhancing their translational relevance [14,15].

This review comprehensively outlines the current understanding of CAVD pathogenesis, the development and application of pertinent animal models, and the methodologies for assessing disease progression within these models. Finally, we highlight the existing challenges and future directions for utilizing animal models in CAVD research, providing guidance to advance this critical field.

## 2. Overview of the Mechanisms of CAVD Development

**Figure 1:**
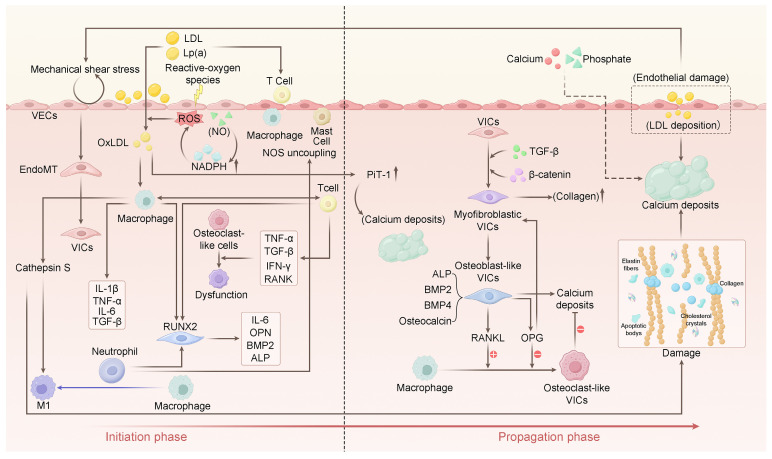
Phases and Key Mechanisms of CAVD Pathogenesis. Initiation Phase: High-velocity blood flow induces endothelial damage, promoting inflammation through increased expression of adhesion molecules, lipid deposition, and macrophage recruitment. Oxidized lipids (Ox-LDL) enhance calcification via upregulation of PiT-1 and pro-inflammatory polarization of macrophages. Endothelial dysfunction leads to reduced eNOS activity. Inflammatory cytokines (TNF-α, IL-6) drive endothelial-to-mesenchymal transition (Endo-MT) via NF-κB signaling, generating valve interstitial cells (VICs). Propagation Phase: Factors derived from M1 macrophages (M1-polarized macrophages) promote osteogenic differentiation of VICs, upregulating osteogenic markers such as OPN, BMP2, BMP4, and ALP. Valve thickening results from calcification and fibrosis. Activated VICs—differentiated into myofibroblast-like and osteoblast-like phenotypes—release calcifying vesicles or undergo apoptosis/necrosis, forming hydroxyapatite nuclei that develop into calcified nodules. Although monocyte-derived osteoclast-like cells can resorb calcification via OPG/RANKL/RANK signaling, their function is impaired by inflammatory cytokines (TNF-α, IFN-γ), disrupting calcification balance and exacerbating mineral deposition.

Although the pathogenesis of CAVD remains incompletely understood, current evidence indicates its development involves several key processes: impaired endothelial barrier function, lipid deposition, immune cell infiltration, and dysregulation of phosphorus and calcium metabolism [3,16,17]. These processes collectively establish a pro-inflammatory and osteogenic microenvironment within the valve. Within this milieu, VICs progressively acquire myofibroblastic and osteoblast-like phenotypes. This phenotypic shift drives fibrocalcific remodeling of the leaflets, ultimately culminating in AS and consequent left ventricular outflow obstruction [1,18] (Figure 1).

### 2.1. Initiation Phase

The pathological initiation phase of CAVD is characterized by lipid deposition, endothelial damage, and inflammatory infiltration. Maintaining the integrity of the aortic valve endothelial layer is essential for normal valvular homeostasis and physiological function [19]. Abnormal hemodynamic forces, particularly high-velocity blood flow generating shear stress, can mechanically disrupt this endothelial layer, compromising its integrity and barrier function. Conversely, oscillatory shear stress on the aortic-facing side can initiate endothelial–mesenchymal transition (Endo-MT) [20,21,22]. Collectively, this endothelial dysfunction establishes a permissive environment for subsequent lipid deposition and immune cell infiltration [23,24].

Lipoprotein deposition represents a significant inducer of chronic inflammation in CAVD. This is supported by fluorodeoxyglucose positron emission tomography–computed tomography (FDG-PET/CT) imaging demonstrating inflammation in aortic valves during the pre-calcification phase [25]. The primary lipoprotein components deposited within valves include ApoB, ApoA, and Apo(a) [26]. Concurrently prevalent in CAVD is dysfunction of the endothelial nitric oxide synthase (eNOS) pathway, alongside valvular deposition of oxidized nicotinamide adenine dinucleotide (NAD) [27,28]. eNOS dysregulation, coupled with elevated nicotinamide adenine dinucleotide phosphate (NADPH) oxidase levels, drives increased production of reactive oxygen species (ROS). The resulting oxidative stress promotes substantial formation of oxidized low-density lipoprotein (ox-LDL) [29]. Ox-LDL deposition subsequently induces an inflammatory response in the valve. This response is characterized by the upregulation of cell adhesion molecules, including intercellular adhesion molecule 1 (ICAM-1) and vascular cell adhesion molecule 1 (VCAM-1) [26,30,31,32], which promotes the adhesion of immune cells. Additionally, Ox-LDL enhances calcification through upregulation of PiT-1 and promotes pro-inflammatory polarization of macrophages (M1 macrophages) [33].

Following phagocytosis by infiltrating macrophages, these lipids stimulate macrophage secretion of tumor necrosis factor-alpha (TNF-α). TNF-α activates nuclear factor kappa-light-chain-enhancer of activated B cells (NF-κB), interleukin-1 beta (IL-1β), and interleukin-6 (IL-6), thereby promoting the osteogenic differentiation of VICs [34,35,36,37,38]. Concurrently, endothelial dysfunction and inflammatory responses induce local cell death, releasing apoptotic vesicles and initiating the formation of localized microcalcifications within the valve. Macrophages and VICs release extracellular vesicles (EVs), which are rich in calcium and inorganic phosphorus. The subsequent formation of hydroxyapatite (HA) crystals within these EVs leads to their rupture [39,40,41,42]. The released HA crystals interact with the extracellular matrix (ECM), exacerbating microcalcification and establishing nucleation sites for further mineralization [39,40]. These mineralization foci, in turn, amplify the inflammatory response, creating a self-perpetuating cycle that drives CAVD progression.

### 2.2. Propagation Phase

Osteogenic differentiation and calcification represent the hallmark features of the propagation phase in CAVD [43]. Following the initiation phase—characterized by chronic inflammatory stimulation and lipid deposition—CAVD progression undergoes a shift, with calcification becoming the predominant pathological alteration [18]. At this stage, calcification is stimulated not only by immune cells but also regulated through multiple signaling pathways. Anatomically, the aortic valve comprises three distinct layers: the fibrosa (aortic-facing), the spongiosa (middle), and the ventricularis (ventricular-facing) [13,44]. A comprehensive proteomic analysis of all three layers by Aikawa et al. revealed heightened signaling activity associated with collagen biosynthesis, modification, and extracellular matrix degradation in VICs residing within the fibrosa layer [45], indicating a heightened propensity for calcification in this region.

The differentiation of VICs into myofibroblasts and osteoblast-like cells represents a critical mechanism in the calcification process during CAVD progression. This process is accompanied by the upregulation of osteogenic markers, including Osteopontin (OPN), Bone morphogenetic protein-2 (BMP2), Bone morphogenetic protein-4 (BMP4), and alkaline phosphatase (ALP) [46,47,48,49]. This process is widely regulated by multiple factors, including cytokines from immune cells [11]. In vitro, transforming growth factor-beta 1 (TGF-β1) stimulates VIC differentiation toward myofibroblasts, whereas fibroblast growth factor-2 (FGF-2) inhibits this process [50]. Myofibroblast differentiation is characterized by increased expression of α-smooth muscle actin (α-SMA), vimentin, and smooth muscle myosin [51]. Subsequently, activation of the mitogen-activated protein kinase (MAPK) pathway promotes the association of low-density lipoprotein (LDL) with glycosaminoglycans, facilitating nodule formation [52,53]. Valvular calcium deposition occurs primarily via two mechanisms: osteogenic differentiation and dystrophic calcification. VIC osteogenic differentiation plays a significant role in the osteogenic pathway [45]. Several key molecular signaling pathways are highly associated with this osteogenic differentiation, including the RANK/RANKL/OPG, Wnt/β-catenin, and Notch1 pathways [6]. The RANK/RANKL/OPG pathway promotes VIC differentiation toward osteoblasts, critically contributing to aortic valve calcification [54]. Binding of Wnt ligands to LRP5 activates the canonical Wnt/β-catenin pathway, which positively regulates osteoblastic differentiation of valve cells and promotes calcification [55]. BMP-2 is a potent inducer of osteoblast differentiation, while Runt-related transcription factor 2 (Runx2) serves as a key transcriptional regulator of osteogenesis. Functional Notch1 signaling inhibits both BMP-2 expression and Runx2 transcriptional activity, thereby suppressing osteoblastic differentiation of valvular mesenchymal stromal cells [56]. Additionally, monocyte-derived osteoclast-like cells are capable of resorbing calcified deposits through the RANK/RANKL/OPG pathway. However, inflammatory cytokines such as TNF-α and IFN-γ impair this osteoclast function, disrupting calcification balance and promoting further deposition [57].

However, osteogenic differentiation contributes to only approximately 13% of calcification in tricuspid aortic valves with advanced aortic stenosis [58]. In contrast, the majority (approximately 83%) of calcific deposits in advanced calcific aortic valves are dystrophic in origin [59]. Some studies suggest this predominance may result from diffuse calcium precipitation on cellular debris following apoptosis of VICs [60]. It should be noted, however, that certain variability exists across studies, and the reported proportions may differ depending on sample characteristics, detection methods, and definition criteria.

Despite substantial advances in related research, the mechanisms driving valvular calcification remain incompletely understood. Consequently, no validated therapeutic target demonstrating clinically meaningful anti-calcification efficacy has yet been identified.

## 3. CAVD Research Animal Models

**Figure 2:**
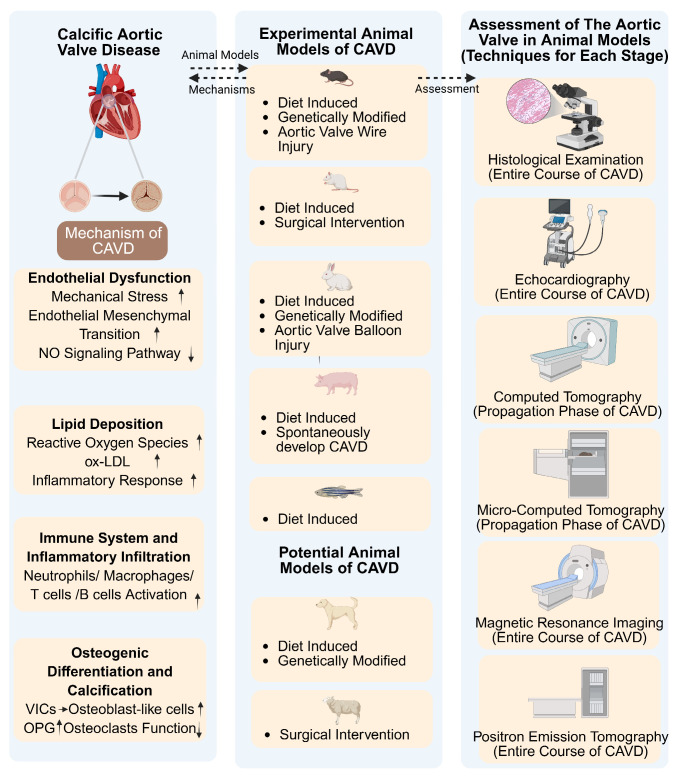
In Vivo Animal Models for CAVD: Pathogenesis, Research Applications, and Detection Techniques. Diverse large and small animal models of CAVD—including spontaneous, induced, and genetically modified models targeting risk factors—have been utilized to elucidate the pathophysiological mechanisms underlying CAVD. Specific models show significant promise for future CAVD research and the evaluation of novel detection technologies.

Interactions among VECs, VICs, and immune cells play a critical role in aortic valve leaflet remodeling and the pathogenesis of CAVD. This complex pathological process is further modulated by systemic factors, including the host’s metabolic status and immune response [18]. To faithfully recapitulate CAVD progression within a living organism, reliable animal models are indispensable for investigating disease mechanisms, therapeutic efficacy, and toxicity. Although species such as mice, rats, rabbits, and swine are commonly employed in CAVD research, no single model has emerged as optimal. The following sections provide an overview of frequently used animal models and those exhibiting potential for modeling CAVD (Figure 2).

### 3.1. Mice Model

The mouse model is extensively utilized for in vivo CAVD research, owing to its cost-effectiveness, high reproductive capacity, and amenability to genetic manipulation. C57BL/6 mice, with a typical lifespan of 18–24 months, reach an age considered equivalent to human senescence around 180 days post-birth, thus serving as a suitable model for investigating the role of aging in CAVD [61]. Although CAVD does not develop spontaneously in mice, it can be induced by feeding a high-cholesterol diet for periods exceeding 6 months [62]. While wild-type C57BL/6 mice typically develop only mild to moderate aortic stenosis under these dietary conditions, employing genetically modified mice (e.g., specific knockouts) effectively overcomes this limitation and promotes more robust calcification [63].

Current experimental mouse models for CAVD research predominantly feature single-gene mutations disrupting lipid metabolism, notably low-density lipoprotein receptor-deficient (Ldlr^−/−^) and apolipoprotein E-deficient (Apoe^−/−^) mice [64]. These models exhibit markedly elevated plasma levels of very low-density lipoprotein (VLDL) and low-density lipoprotein (LDL). When challenged with a high-cholesterol diet, they readily develop significant aortic valve calcification and initial CAVD manifestations. The Ldlr^−/−^ and Apoe^−/−^ models display distinct phenotypic characteristics. In Ldlr^−/−^ mice, plasma cholesterol is primarily LDL-associated, offering a more human-like lipid profile. Pathological changes in this model are primarily driven by hyperlipidemia, as LDLR deficiency minimally impacts systemic inflammation. However, Ldlr^−/−^ mice on a high-cholesterol diet are susceptible to metabolic syndrome, potentially confounding study endpoints [65]. Conversely, Apoe^−/−^ mice spontaneously develop hypercholesterolemia even on a normal diet and exhibit a propensity for metabolic syndrome. Lipid streaks appear in the proximal aorta by 3 months of age, and diet-induced cardiovascular injury severity correlates with cholesterol intake [66]. Importantly, the plasma lipoprotein profile of Apoe^−/−^ mice diverges from humans: most cholesterol is carried by VLDL and chylomicrons (not LDL), limiting its fidelity to human CAVD progression [67,68]. While both models frequently develop aortic leaflet calcification due to lipid deposition and inflammation, significant hemodynamic stenosis is uncommon [14]. The introduction of the double-mutant hypercholesterolemic model (LDLR^−/−^/ApoB^100/100^) effectively addresses this limitation. This model develops significant hemodynamic stenosis and represents a more suitable platform for mechanistic studies [64,69]. Feeding this double-mutant model a high-cholesterol diet for 12 months significantly increases hemodynamic stenosis incidence, enabling exploration of specific mechanisms, such as activated platelets accelerating aortic valve calcification [70].

Rattazzi et al. demonstrated that adding warfarin or rivaroxaban to the Western diet of Apoe^−/−^ mice differentially influenced aortic valve calcification in this model of CAVD with dyslipidemia [71]. Histological and ultrasonographic analyses after 8 weeks revealed significantly increased calcification in the aortic valves of warfarin-treated mice compared to controls, whereas rivaroxaban treatment showed no significant difference. This suggests warfarin may facilitate establishing a CAVD model in this setting. Underlying dyslipidemia provides the foundation for this model. Conversely, in the Ldlr^−/−^ ApoB100^APOB100^*/*^APOB100^ mouse model, dietary supplementation with menaquinone-4 (MK-4) increased plasma lipid levels but did not affect aortic valve morphology, calcification, or fibrosis [72]. Strategically utilizing drugs that promote aortic valve calcification represents a promising approach for developing optimized CAVD animal models.

A significant limitation of Apoe^−/−^ mice is their poor ability to finely modulate plasma cholesterol levels and atherosclerosis extent. More critically, these mice respond poorly to established lipid-lowering and anti-atherosclerotic drugs, restricting their utility for drug efficacy testing [73]. In contrast, transgenic mice expressing both human mutant APOE (APOE3-Leiden) and human cholesteryl ester transfer protein (CETP), designated APOE3-Leiden CETP mice, exhibit moderate, modifiable hyperlipoproteinemic and atherosclerotic phenotypes. These mice demonstrate favorable responses to lipid-lowering and anti-atherosclerotic therapies [74]. Hyperlipidemia-induced lipid deposition constitutes a key pathological feature in early CAVD [75]. Supporting this, Carracedo et al. observed valve thickening after 16 weeks of Western diet feeding; notably, however, alizarin red and von Kossa staining for calcification remained negative [76]. This finding is consistent with early-stage CAVD pathological changes. Consequently, the APOE3-Leiden.CETP model holds promise as a valuable tool for evaluating the therapeutic effects of lipid-lowering and anti-atherosclerotic drugs on early CAVD.

A novel C57BL/6J mouse model for atherosclerosis research has recently been developed through adeno-associated virus serotype 8 (AAV8)-mediated delivery of the gain-of-function mutant Pcsk9^D377y^ [77]. Proprotein convertase subtilisin/kexin type 9 (PCSK9), predominantly expressed in the liver, binds to the LDLR, promoting its lysosomal degradation in hepatocytes [78]. The D377Y mutation (aspartic acid substitution for tyrosine at residue 377) confers a gain-of-function phenotype. AAV8 efficiently transduces hepatocytes, leading to hepatic overexpression of this mutant PCSK9, enhanced LDLR degradation, and consequent hyperlipidemia [79]. This model shares phenotypic similarities with Ldlr^−/−^ mice but enables rapid generation of hyperlipidemic mice without the need for cross-breeding (e.g., with Apoe^−/−^ or Ldlr^−/−^ strains), thereby accelerating atherosclerosis research. Although not yet utilized in CAVD studies, the shared pathophysiological underpinnings of CAVD and atherosclerosis suggest potential applicability of this model for CAVD research. Validating its utility for CAVD modeling will require comprehensive hemodynamic assessment and detailed histopathological analysis of aortic valve tissues.

Beyond lipid-centric models, several transgenic mouse models exhibit key pathological features of CAVD, including aortic valve leaflet calcification, even when maintained on a standard diet. These models provide valuable opportunities to investigate CAVD pathogenesis in vivo independent of high-fat dietary influences, offering a more precise simulation of the disease’s complex initiation characteristics. Notable gene-modified models include mice deficient in chondromodulin-I (Chm-I; Chm1^−/−^), C-X-C chemokine receptor type 4 (CXCR4; Cxcr4^−/−^), recombination signal binding protein for immunoglobulin kappa J region (RBPJκ; Rbpj^−/−^), palmdelphin (Palmd; Palmd^−/−^), Klotho (Klotho ^−/−^), interleukin-1 receptor antagonist (IL-1Ra; Il1rn^−/−^), and mitsugumin 53 (MG53; Trim72^−/−^) [80,81,82,83,84,85,86].

Aged Chm1^−/−^ mice (average ~90 weeks) exhibit typical pathological features of aortic stenosis progression, including early valvular lesions, inflammatory cell infiltration, lipid deposition, calcification, and notably, neoangiogenesis—a feature less common in other murine CAVD models [80]. While most CAVD mouse models require prolonged induction periods (often >10–20 weeks), significant aortic stenosis develops rapidly in male endothelial-specific Cxcr4 knockout mice (Tie2-Cre/Cxcr4^f1/f1^) fed a normal diet, detectable by echocardiography at 6 weeks; females develop stenosis even faster, within 3 weeks [81]. Aortic valve calcific nodules in Klotho-deficient (Kl^−/−^) mice closely resemble those in human CAVD [84]. This similarity stems primarily from the development of kidney disease and elevated serum phosphate levels in this model, both established human CAVD risk factors [87]. Furthermore, minimal immune cell infiltration in the valves allows focused investigation of VIC-specific molecular changes. Although echocardiography showed no difference in peak aortic valve velocity between 10-month-old Trim72^−/−^ (MG53-deficient) and wild-type (WT) mice, the mean peak velocity in Trim72^−/−^ mice increased significantly from 1274 ± 66.3 cm/s at 10 months to 2929 ± 1073 cm/s at 24 months (Δv vs. WT, *p* < 0.0001), providing hemodynamic evidence of progressive aortic valve disease [86]. Crucially, Trim72^−/−^ mice exhibit age-dependent CAVD onset without baseline alterations in ventricular systolic function, establishing this model as significant for CAVD research.

Identified gene mutations in specific mouse models correlate with congenital aortic valve developmental defects in humans, contributing to an elevated incidence of CAVD. As the most prevalent congenital heart defect, bicuspid aortic valve (BAV) affects approximately 1–2% of the population [88]. Notably, 30–50% of patients undergoing surgery for CAVD present with BAV, with disease onset occurring on average a decade earlier than in those with tricuspid aortic valves [89]. Studies indicate that BAV has a heritability as high as 89%, suggesting a strong genetic basis in most cases. Although BAV is widely recognized as heritable, its inheritance does not follow a single-gene model. Key genes implicated in murine BAV development include endothelial nitric oxide synthase (Nos3), notch receptor 1 (Notch1), natriuretic peptide receptor 2 (Npr2), and discoidin, CUB and LCCL domain-containing 2 (Dcbld2) [90,91,92,93]. Phenotypic penetrance and expressivity in these models vary considerably depending on genetic background, reflecting heterogeneity observed in human BAV patients. For instance, Nos3^−/−^ mice on a C57BL/6J background exhibit BAV penetrance ranging from 27% to 42% [90,94,95]. Similarly, Notch1^+/−^ models show a BAV penetrance of 64% [96]. Only approximately 9.4% of Npr2^−/−^ mice develop BAV [92]. Dcbld2^−/−^ mice spontaneously develop aortic valve calcification and hemodynamically significant aortic stenosis (AS), with about 50% exhibiting BAV by one year of age [93]. BAV is the most common congenital cardiac malformation and exhibits a male predominance with a ratio of approximately 3:1 [97]. Consistently, Gata6^+/−^ mice show a similar sex-biased penetrance, affecting 56% of males and 27% of females [98].

While established dietary and transgenic methods exist for modeling cardiovascular diseases, emerging interest focuses on novel animal models utilizing direct mechanical injury to the aortic valve. This technique involves echocardiography-guided insertion of a guidewire via the right common carotid artery into the left ventricle, followed by intentional scratching of the aortic valve leaflets to induce localized injury [99]. Niepmann et al. recently refined this model by modulating the extent of valve damage to recapitulate distinct stages of CAVD pathology [100]. This approach enables stage-specific investigation of CAVD progression, reliably producing key pathological hallmarks—including inflammation, oxidative stress, apoptosis, leaflet thickening, fibrosis, calcification, hemodynamically significant AS, and aortic regurgitation (AR)—thereby mirroring common clinical manifestations. Nevertheless, while the guidewire injury model robustly simulates human CAVD, it inherently models acute injury rather than the decades-long chronic mechanical stress central to spontaneous human disease (Table 1).

### 3.2. Rat Model

Although mice offer greater ease for genetic manipulation in generating specific knockout or overexpression models, rats exhibit closer physiological, morphological, and genetic similarities to humans, rendering them ideal for biomedical and clinical research [103,104]. In cardiovascular studies, rats display a weaker response to dietary cholesterol than mice. However, atherogenic diets (supplemented with bile acids or thiouracil) effectively induce hyperlipidemia and atherosclerosis in rats, better recapitulating human-like lipid deposition processes [105]. Reaves et al. developed Ldlr^−/−^ hamsters exhibiting impaired clearance of circulating large lipoproteins and accelerated atherosclerosis [106]. Zeng et al. subsequently demonstrated that this model develops significant valvular calcification after 16 weeks on a Western diet, though without concomitant hemodynamic manifestations of AS. Beyond lipid metabolism gene editing, rat CAVD models can also be established by targeting microRNAs (miRNAs), a well-established class of non-coding RNAs. Xu et al. successfully generated miR-214 knockout rats. When challenged with a Western diet and intraperitoneal vitamin D injections for 16 weeks, these miR-214^−/−^ rats exhibited aortic valve thickening, calcification, and increased transvalvular velocity [107,108].

Similar to murine CAVD models, rats can develop CAVD through pharmacological induction, though surgical approaches are also utilized. In rat-based CAVD research, warfarin administration—either via diet or subcutaneous injection—is the primary method for inducing aortic valve calcification [109]. To prevent life-threatening coagulopathy, concurrent vitamin K1 supplementation is essential. Notably, studies by Husseini et al. indicate that warfarin-induced models may develop AS [110]. Alternatively, vitamin D supplementation for 10 weeks induces characteristic CAVD clinical signs and hemodynamic alterations in rats. However, this approach exclusively promotes aortic valve and vascular calcification without concurrent AS development [111].

Metabolic disorders associated with chronic kidney disease (CKD) constitute significant risk factors for CAVD development [87]. To investigate CKD-mediated pathology, Shuvy et al. established a Sprague-Dawley rat model using a phosphate-enriched, uremia-inducing diet. This model recapitulated aortic valve calcification linked to inflammation and osteoblastic activity, as validated by histology, echocardiography, and computed tomography. Notably, calcification regressed upon dietary cessation and mineral balance restoration, demonstrating its dynamic reversibility [112]. Complementing this approach, Messaoudi et al. developed a surgical CKD model in rats involving bilateral renal artery ligation and renal tissue electrocautery, combined with a high-phosphorus diet—a feasible strategy given rodents’ surgical accessibility [113]. This model provides a robust platform for delineating the pathogenesis of CKD-associated CAVD.

Advancing gene editing technologies and increasingly comprehensive rat genomic mapping are poised to drive the development of numerous rat-specific gene-targeted models in the near future. This progress is expected to yield tailored rat models of CAVD, capable of catering to the diverse research demands of the field (Table 2).

### 3.3. Rabbit Model

The rabbit model is widely utilized in CAVD research owing to its anatomical similarities to humans, including a tri-leaflet aortic valve structure. Furthermore, rabbits exhibit a plasma lipoprotein profile similar to humans, rendering them a valuable model for investigating the role of dyslipidemia in CAVD pathogenesis [114].

New Zealand White rabbits represent the predominant strain for modeling CAVD. These models typically employ a high-cholesterol diet supplemented with vitamin D_2_ to induce severe AS, detectable within 40 weeks [115]. The critical role of vitamin D_2_ was definitively established by Doan et al. in a controlled trial [116]; rabbits fed this diet for 12 weeks developed significant aortic valve calcification, reduced valve area, and clinical AS signs, directly linking vitamin D_2_-induced hypercalcemia to CAVD pathogenesis. However, high-cholesterol diets frequently cause hepatic impairment in rabbits, leading to low survival rates [117]. To circumvent this limitation, Lu et al. generated LDLR^−/−^ rabbits via knockout of the LDL receptor gene’s protein-coding region [118]. This model spontaneously develops hypercholesterolemia without dietary manipulation, eliminating diet-associated confounders.

Although the aforementioned gene-edited rabbits have not yet been applied to CAVD research, Watanabe Heritable Hyperlipidemic (WHHL) rabbits—possessing analogous mutations—are extensively studied in this field. Beyond sharing the advantages of LDLR^−/−^ rabbits, WHHL rabbits fed a standard diet spontaneously develop aortic stenosis, calcification, and upregulation of calcification-associated gene pathways [117]. Moreover, these pathologies exhibit clear age-dependent progression in WHHL rabbits, significantly advancing our understanding of age as a critical risk factor for CAVD development (Table 3).

### 3.4. Swine Model

The porcine cardiovascular system exhibits significant anatomical and morphological homology with humans, particularly in aortic valve structure. Similarly to human valves, porcine aortic valves possess a tricomposite layered architecture. Unlike murine and rabbit models, swine spontaneously develop valvular sclerosis lesions [120]. Genomic analyses reveal closer evolutionary proximity between swine and humans compared to rodent models, suggesting superior translational relevance at the molecular level, highlighting swine’s potential for CAVD research [121]. Zabirnyk et al. established an ex vivo model using cultured intact porcine valve leaflets, maintaining structural integrity while enabling observation of calcification under pro-calcific conditions [122]. Complementing this, Sider et al. conducted histopathological and immunohistochemical analyses on Yorkshire swine fed high-fat diets. After 5 months, these swine developed proteoglycan-enriched fibrotic lesions in aortic valves—an early hallmark of CAVD pathogenesis [120]. These findings demonstrate remarkable conservation of early CAVD pathological features between swine and humans, positioning porcine models as ideal platforms for investigating initial disease mechanisms.

Early CAVD pathological features—including leaflet thickening, oxidized lipid accumulation, and macrophage infiltration—are evident in Rapacz familial hypercholesterolemic swine [123]. During this initial phase, valvular function remains largely preserved, with advanced aortic stenosis requiring additional pathogenic stimuli. Sider et al. have suggested extending the use of established porcine atherosclerosis models harboring lipid metabolism mutations to CAVD research [124]. Currently, analogous to murine models, swine with ApoE, LDLR, and double-knockout mutations are primarily utilized in atherosclerosis studies [125]. Consequently, dedicated CAVD validation studies are warranted for these porcine models (Table 4).

### 3.5. Canine Model

Canine models are valuable for cardiovascular disease research due to their physiological similarities with humans, particularly in metabolic processes, cardiovascular structure/function, and other systems. While surgical methods have established canine models of aortic stenosis, these models typically lack pathological changes in the aortic valve [126]. Consequently, canine models have not been employed in studies specifically investigating CAVD.

Canine models can spontaneously develop atherosclerotic lesions similar to those in humans, accompanied by analogous clinical complications [127]. Atherosclerosis in these models can be exacerbated by a high-fat, high-cholesterol diet or by inducing mutations in lipid metabolism genes. Research indicates that adipokines contribute to acute endothelial dysfunction by impairing endothelial vasodilatory mechanisms. This suggests that chronic exposure to circulating adipokines, derived from adipose tissue, may lead to systemic endothelial dysfunction. Given the shared pathogenesis between early atherosclerosis and CAVD, the canine atherosclerosis model might also exhibit pathological changes in the aortic valve; however, this hypothesis requires further validation through in-depth research. Notably, distinct differences exist in the plasma lipid profiles of dogs and humans. Canine blood cholesterol is predominantly composed of high-density lipoprotein (HDL) particles, resembling rodents, whereas human blood cholesterol is primarily made up of LDL particles [128]. Moreover, as companion animals, canines raise unique ethical concerns, and their lower societal acceptance limits their use in CAVD research in many countries [129,130].

### 3.6. Other Animal Models

Various animal models serve as valuable tools for studying cardiovascular diseases, each offering distinct advantages for research. For instance, zebrafish, owing to their similarity to mammals in heart development, are frequently employed to investigate cardiac development and disease mechanisms [131]. Notably, Gollmann et al. even identified a molecular pathway associated with CAVD through studies of zebrafish mandibular formation [132]. Conversely, sheep share significant similarities with humans in cardiovascular anatomy and physiology, making them a preferred model for surgical procedures [133,134]. Consequently, sheep are extensively used in preclinical evaluations of artificial heart valve replacements, providing a model that closely recapitulates the pathophysiological changes observed in human cardiovascular diseases.

## 4. Histologic and Imaging Assessment of the Aortic Valve in Animal Models of CAVD

Animal models are crucial for investigating CAVD mechanisms, enabling comprehensive analysis of aortic valves using reliable assays. While histopathology provides a straightforward and reliable assessment, it does not allow for longitudinal monitoring. Consequently, multimodal imaging approaches have become widely used to evaluate aortic valve characteristics and calcification in vivo.

### 4.1. Histological Examination of Aortic Valve in Animal Models

Histologic examination of the aortic valve is essential for accurately assessing valvular morphology and quantifying calcium deposition in animal models [135]. Commonly used techniques for detecting valve calcification include Alizarin red staining, which binds calcium ions, and von Kossa staining, which reacts with carbonates and phosphates [135,136]. While both assays provide insight into the degree of calcification, Alizarin red staining has been shown to be more effective than von Kossa staining for detecting calcification in C57BL/6 mouse aortic valves, where pigmented deposits can interfere with interpretation due to similar coloration. Overall, von Kossa staining exhibits greater sensitivity for calcified lesions, whereas Alizarin red offers higher specificity. Therefore, their combined use is recommended for more accurate assessment of calcification. Beyond calcification, analysis of other valve tissue components is crucial. Collagen can be assessed using Masson’s trichrome or Picrosirius red staining, while Movat’s pentachrome stain allows simultaneous visualization of multiple components, including collagen, elastin, and proteoglycans [137,138]. Furthermore, attention should be paid to the valve commissures, as calcification commonly initiates at these attachment points.

### 4.2. Echocardiography

Echocardiography, a non-invasive, safe, and cost-effective modality, is a key tool for assessing heart valve function and is commonly employed in CAVD animal models. Key indicators for evaluating aortic stenosis include aortic valve area, mean pressure gradient, and peak flow velocity, which effectively reflect valvular function and hemodynamic alterations. Although echocardiography cannot precisely quantify calcium levels, it remains the gold standard for assessing valve function, degenerative changes (including fibrotic leaflet thickening and calcification), and overall stenosis severity [139]. For murine cardiac ultrasound imaging, studies have recommended the use of 30 MHz probes (e.g., VisualSonics Vevo 770) for examination. Prior to imaging, the chest hair should be thoroughly removed to minimize acoustic interference. Throughout the procedure, heart rate and core body temperature must be continuously monitored to reduce the impact of anesthesia on cardiac function. It is generally advised to maintain the heart rate between 400 and 500 beats per minute and body temperature at 37 °C [140]. Aortic valve peak velocity is measured in the suprasternal view using pulse-wave Doppler with angle correction set between 45° and 55° [141]. Currently, there is no universally accepted criterion for grading aortic stenosis in mice based on this parameter. Some studies define mild, moderate, and severe stenosis as a peak velocity increase of 15–50%, 50–75%, and greater than 75% above baseline, respectively [100]. In contrast, other researchers consider an absolute aortic valve peak velocity exceeding 2000 mm/s as indicative of stenosis [142]. Compared to transthoracic echocardiography (TTE), transesophageal echocardiography (TEE) provides superior image clarity in large-animal models, particularly for differentiating between tricuspid and bicuspid aortic valves [143].

### 4.3. Computed Tomography

Computed Tomography (CT) is widely used in clinical practice to identify and quantify aortic valve calcification. The calcium score, calculated using the Agatston method based on the density and volume of calcified plaques detected by CT, shows a strong correlation with the weight of excised calcified valves [144]. This score reflects the progression of CAVD and correlates strongly with the severity of aortic stenosis as assessed by echocardiography. Therefore, CT serves as a valuable tool for the quantitative evaluation of aortic valve calcification in large animal models. However, CT has several limitations. For example, in young patients with early-stage (diastolic) CAVD, the mean transaortic pressure gradient may not correlate with calcium density, reducing the accuracy of CT-based calcification assessment in this population [145]. Studies also indicate that CT-assessed calcium burden in CAVD may differ by sex [146]. Furthermore, CT exhibits limited sensitivity in detecting calcification, often failing to visualize early microcalcifications and only revealing larger, confluent calcified areas. Nevertheless, recent research has demonstrated that contrast-enhanced CT can not only evaluate the extent of calcification but also detect the non-calcified (fibrotic) component of the aortic valve, which is characteristic of early CAVD [147]. Thus, CT represents a promising technique for assessing valvular calcification in large animal models.

### 4.4. Micro-Computed Tomography

Micro-Computed Tomography (Micro-CT) enables high-resolution analysis of the morphology and mineral density within excised aortic valves. With a spatial resolution reaching 1 µm, Micro-CT is ideally suited for quantifying structural parameters such as porosity, bone thickness, density, particle diameter, and fiber orientation [148]. The technique relies on X-ray attenuation, which varies with material density and thickness, allowing differentiation between calcified and soft tissues. This capability facilitates the generation of contrast-enhanced images depicting calcification distribution and enables precise volumetric quantification of calcified tissue, which correlates with aortic stenosis severity [149]. Compared to the Agatston score, this method offers superior reproducibility, greater stability, and improved sensitivity for detecting small calcified lesions, making it particularly suitable for use in small animal models [150,151]. Therefore, Micro-CT can be applied for in vivo imaging in small animal models. For instance, Gollmann et al. utilized Micro-CT to analyze the relationship between aortic valve calcification and bone formation, demonstrating its utility through concurrent assessment of femur alterations in mice [132]. Spelsberg et al. employed a Scanco Micro-CT 40 system to evaluate the presence and extent of calcification in male New Zealand white rabbits. Scanning parameters included 45 kV voltage, approximately 8 μm voxel size, and optimized sensitivity (1000 projections, 2048 samples, 0.3 s integration time per projection) [152]. The high resolution of micro-CT also allows detailed imaging and characterization of pathological changes in aortic valves in small animal models; however, care should be taken to differentiate such changes from calcification occurring in the mouse aortic arch [153].

### 4.5. Magnetic Resonance Imaging

Compared to CT, Magnetic Resonance Imaging (MRI) is less commonly employed for diagnosing and managing CAVD. However, it serves as a valuable complementary imaging modality, particularly in aortic stenosis [154]. MRI enables assessment of valve anatomy, flow quantification, and left ventricular volume, mass, remodeling, and function. Its precise anatomical analysis contributes to a growing role in planning aortic valve replacement surgery. The latest consensus document from the European Society of Cardiovascular Radiology highlights potential advantages of MRI, including comprehensive measurements of the valve and aortic root, assessment of ventricular function, and evaluation of the aorta [155]. MRI offers benefits over invasive tests such as TEE and cardiac catheterization and avoids contrast agent use in cases of severe renal impairment [156]. Despite this limitation, MRI cannot assess the degree of aortic valve calcification. In recent years, 4D Flow MRI has emerged as a novel vascular imaging technique. Unlike conventional 3D vascular imaging, it incorporates temporal resolution (time-resolved), allowing visualization of hemodynamic changes throughout the cardiac cycle [157]. Moreover, 4D Flow MRI serves as a key technique linking valvular morphology and hemodynamic abnormalities to pathological changes in the aortic wall. It not only provides deeper insights into the mechanisms underlying aortic pathologies but also offers promising imaging-based evidence for hemodynamic evaluation in large animal models of CAVD [158].

### 4.6. Positron Emission Tomography (PET)

Positron emission tomography (PET) utilizes radioactive tracers that accumulate in areas of high metabolic activity, enabling the detection of molecular and cellular changes before observable anatomical alterations occur. Currently, two primary tracers are used in PET imaging: ^18^F-fluorodeoxyglucose (^18^F-FDG) and ^18^F-sodium fluoride (^18^F-NaF) [159]. ^18^F-FDG, a glucose analog, accumulates in metabolically active cells and serves as a sensitive marker for valvular inflammation. It is taken up by cells via glucose transporter proteins (GLUTs), reflecting the glucose uptake process in macrophages and other inflammatory cells. The enhanced glycolytic activity in these cells makes ^18^F-FDG a suitable target for inflammation imaging with PET [160,161]. Multiple studies have confirmed a correlation between ^18^F-FDG signal and atherosclerotic plaques, supporting its role as an early indicator of CAVD. This is further emphasized by the colocalization of macrophage-rich regions with ^18^F-FDG–positive areas [160,162,163]. In contrast, ^18^F-NaF is a bone-seeking tracer that binds to hydroxyapatite, a key component of valvular calcification, reflecting its extent. Notably, some studies suggest that ^18^F-NaF PET/CT may predict hemodynamic progression in calcific AS, whereas ^18^F-FDG PET/CT does not significantly predict the development of severe AS [164]. This discrepancy likely arises because hemodynamic progression in AS is primarily driven by valve calcification. Supporting this, Sadeghi et al. employed ^18^F-NaF PET in a mouse model of CAVD, demonstrating through both PET and autoradiography an age-dependent increase in aortic valve microcalcification, indicating aging as a significant risk factor [93]. Collectively, PET holds significant potential to enhance our understanding of CAVD pathogenesis by elucidating its molecular mechanisms and enabling dynamic in vivo tracking of pathological processes.

## 5. Conclusions and Prospects

Effective treatment of CAVD requires a comprehensive understanding of its etiology and pathogenesis. Despite significant progress over the past two decades in elucidating various pathogenic mechanisms, our knowledge of CAVD pathogenesis remains incomplete. Animal models offer a valuable platform for validating novel CAVD mechanisms and evaluating the efficacy of pharmacological interventions. Current CAVD animal models primarily induce rapid valve calcification through high-cholesterol diets and genetic manipulation of lipid metabolism. Future model development should focus on achieving greater precision by leveraging advanced gene editing technologies and insights from epigenetic regulation. This will enable higher-fidelity recapitulation of human CAVD, including accurate modeling of distinct disease progression stages. For instance, models mimicking the early asymptomatic phase of CAVD would facilitate research into early diagnosis and therapeutic strategies. Similarly, developmental programming models could elucidate how early-life environmental factors influence cardiovascular disease susceptibility, informing preventive approaches. Furthermore, given the multifactorial nature of CAVD, future animal models must emphasize simulating the complex interplay among genetic, environmental, and lifestyle factors, as well as interactions between multiple systems (e.g., endocrine and immune). Developing such integrated models aims to foster a more comprehensive and accurate understanding of CAVD’s intricate pathogenesis.

Animal models provide distinct advantages for research, including the ability to simulate human disease states, shorter lifespans, controllable environments, ethical permissibility, rapid reproductive cycles, and well-defined genetic backgrounds. However, they face inherent challenges due to metabolic, physiological, and anatomical differences compared to humans. Addressing these limitations necessitates a deeper understanding of the underlying pathophysiology and enhancing the translational value of animal models. Strategies include developing humanized models (e.g., humanized mice) and employing genetic engineering to create models with cardiovascular characteristics more closely resembling humans, thereby improving the fidelity of drug screening and evaluation. Moreover, monitoring based on biomarkers or hemodynamic parameters will enable real-time tracking of disease progression, enhance model sensitivity, and provide a more detailed understanding of CAVD formation and developmental stages. This approach allows for a more nuanced understanding of disease dynamics and improves the accuracy and applicability of these therapeutic testing models. Concurrently, the timely integration of novel multi-omics technologies, such as spatial transcriptomics, proteomics, and single-cell genomics, will facilitate the further refinement of CAVD animal models, enabling more precise experimental design.

Ultimately, validating disease mechanisms and bridging preclinical findings to clinical applications depend critically on human patient data and large-scale population studies. Consequently, the future development of animal models for CAVD research will increasingly prioritize precision modeling, simulation of multifactorial interactions and distinct disease stages, and enhancing translational relevance. These advancements aim to provide a stronger scientific foundation for the prevention, diagnosis, and treatment of CAVD.

## Figures and Tables

**Table 1:** Strengths and Limitations of Mouse Models for CAVD. HF: high fat. HC: high cholesterol. Hemodynamically significant stenosis was defined as a ≥50% increase in the peak velocity relative to the baseline value.

Species	Diet	Histopathological Changes in Aortic Valve	Advantages	Limitations	Hemodynamically Significant Stenosis?	Respond to Interventions?
Mice						
ApoE^−/−^	Chow	Lipid deposition Calcification Monocyte/inflammatory cell infiltration	Develop hyperlipidemia and complex lesions on regular chow diet	Absence of a human-like lipid profile	<2% [101]	No
	HF/HC	Lipid deposition Fibrosis Calcification Monocyte/inflammatory cell infiltration	Higher total cholesterol levels than Ldlr^−/−^ mice		<2% [62,102]	Yes
LDLR^−/−^	HF/HC	Lipid deposition Calcification Monocyte/inflammatory cell infiltration	Lipoprotein pattern similar to humans Develop site-specific lesions in a time-dependent manner Ldlr mutations are common in patients with familial hypercholesterolemia	Require HF/HC Diet for CAVD induction	No	Yes
LDLR^−/−^/ApoB^100/100^	Chow	Lipid deposition Calcification Monocyte/inflammatory cell infiltration Myofibroblast activation	Develop CAVD on a regular chow diet Develop more human-like hypercholesterolemia	Expensive and time-consuming	Yes, ~30% [64]	No
	HF/HC	Lipid deposition Calcification Monocyte/inflammatory cell infiltration Myofibroblast activation Fibrosis			Yes, ~50% [70]	Yes
APOE*3-Leiden.CETP	HF/HC	Lipid deposition Fibrosis Thickness	Develop more human-like hypercholesterolemia Favorable response to lipid-lowering and anti-atherosclerotic medications	Absence of positive alizarin red or von Kossa staining	Not known	Yes
Chm1 ^−/−^	Chow	Lipid deposition Thickness Neoangiogenesis Calcification Monocyte/inflammatory cell infiltration	Develop CAVD on a regular chow diet Associated with neoangiogenesis	Expensive and time-consuming	Yes [80]	No
Tie2-Cre/CXCR4^fl/fl^	Chow	Fibrosis Thickness Calcification	Time-saving Develop CAVD on a regular chow diet	Lack of early pathological characterization of CAVD	Yes, 50–90% [81]	No
Klotho^−/−^	Chow	Calcification	Develop CAVD on a regular chow diet Non-Monocyte/inflammatory cell infiltration Develop robust nodular aortic valve calcification similar to the human Develop kidney disease and elevated serum phosphate levels	Expensive and time-consuming Limited scope	Not known	Yes
MG-53^−/−^	Chow	Fibrosis Thickness Calcification	Develop CAVD on a regular chow diet Displayed an age-dependent phenotype of CAVD	Time-consuming	Yes [86]	No
eNOS^−/−^	Chow	Calcification	Bicuspid aortic valves in ~40% of mice	Limited scope	Not known	Yes
NOTCH-1^+/−^	HF/HC	Thickness Calcification	Develop bicuspid aortic valves	Limited scope	No	Yes
NPR-2^+/−^	HF/HC	Fibrosis Thickness Calcification	Bicuspid aortic valves in 9.4% of mice	Limited scope	Yes [92]	No
DCBLD2^−/−^	Chow	Calcification	Bicuspid aortic valves in ~50% of mice	Limited scope	Yes [93]	Yes
C57BL/6J	Chow+ Mechanical injury	Thickness Calcification	Controlled injury Time-saving	Limited relevance to human pathophysiology High mortality rate. Standardization to be improved.	Yes [99]	Yes

**Table 2:** Strengths and Limitations of Rats Models for CAVD. WD: Western diet. Hemodynamically significant stenosis was defined as a ≥50% increase in the peak velocity relative to the baseline value.

Species	Diet	Histopathological Changes in Aortic Valve	Advantages	Limitations	Hemodynamically Significant Stenosis?	Respond to Interventions?
Rat						
LDLR^−/−^	HF/HC	Thickness Calcification	Time-saving Similar to the process of lipid deposition induction in humans	Expensive and time-consuming	No [106]	No
miR-214^−/−^	WD + Vitamin D_2_	Calcification Thickness Monocyte/inflammatory cell infiltration	Time-saving	Expensive and time-consuming	Yes [108]	No
Sprague-Dawley	Rodent diet 5001 + Warfarin + Vitamin K_1_	Calcification	Time-saving	Impaired coagulation.	Not known	No
	Chow + Vitamin D_3_	Calcification	Cheap and easy to operate	Limited relevance to human pathophysiology	No [111]	No
	High-phosphate Diet	Calcification Monocyte/inflammatory cell infiltration	Cheap and easy to operate Associated with chronic renal disease	Limited scope Reversible valve calcification	No [112]	No

**Table 3:** Strengths and Limitations of Rabbits Models for CAVD. HF: high fat. HC: high cholesterol. Hemodynamically significant stenosis was defined as a ≥50% increase in the peak velocity relative to the baseline value.

Species	Diet	Histopathological Changes in Aortic Valve	Advantages	Limitations	Hemodynamically Significant Stenosis?	Respond to Interventions
Rabbit						
New Zealand White	HF/HC + Vitamin D_3_	Lipid deposition Calcification Inflammatory cell infiltration	Time-saving Similar to a plasma lipid protein distribution of humans	Liver dysfunction	Yes [116]	Yes
Watanabe	HF/HC	Lipid deposition Fibrosis Calcification Inflammatory cell infiltration	Time-saving	Require HF/HC Diet for CAVD induction	No [119]	No
	Chow	Thickness Calcification Inflammatory cell infiltration	Develop CAVD on a regular chow diet Age-dependent Spontaneously develop CAVD Avoid liver dysfunction	Time-consuming	Yes [117]	No

**Table 4:** Strengths and Limitations of Swine Models for CAVD. HF: high fat. HC: high cholesterol. Hemodynamically significant stenosis was defined as a ≥50% increase in the peak velocity relative to the baseline value.

Species	Diet	Histopathological Changes in Aortic Valve	Advantages	Limitations	Hemodynamically Significant Stenosis?	Respond to Interventions
Swine						
Yorkshire	HF/HC	Lipid deposition	Develop valvular sclerosis lesions spontaneously	Expensive and time-consuming Lack of late pathological changes in CAVD.	No [120]	No
Rapacz familial hypercholesterolemic	Chow	Thickness Lipid deposition Inflammatory cell infiltration	Few complications Show numerous critical features of early CAVD	Expensive and time-consuming Lack of late pathological changes in CAVD.	No [123]	No

## Abbreviations

ALP	alkaline phosphatase
Apo	apolipoprotein
AS	aortic stenosis
BAV	bicuspid aortic valve
BMP	bone morphogenetic protein
CAVD	calcific aortic valve disease
CKD	chronic kidney disease
CT	computed tomography
ECM	extracellular matrix
Endo-MT	endothelial–mesenchymal transition
eNOS	endothelial nitric oxide synthase
EV	extracellular vesicle
FDG-PET/CT	fluorodeoxyglucose positron emission tomography-computed tomography
ICAM-1	intercellular adhesion molecule 1
IFN-γ	interferon-γ
IL	interleukin
MAPK	mitogen-activated protein kinase
MRI	Magnetic Resonance Imaging
NAD	nicotinamide adenine dinucleotide
NADPH	nicotinamide adenine dinucleotide phosphate
NF-κB	nuclear factor kappa-light-chain-enhancer of activated B cells
OPG	osteoprotegerin
OPN	osteopontin
ox-LDL	oxidized low-density lipoprotein
PCSK9	Proprotein convertase subtilisin/kexin type 9
PiT-1	inorganic phosphate transporter-1
RANKL	receptor activator of nuclear factor κB ligand
ROS	reactive oxygen species
Runx2	Runt-related transcription factor 2
TNF	tumor necrosis factor-alpha
VCAM-1	vascular cell adhesion molecule 1
VECs	valve endothelial cells
VICs	valve interstitial cells
